# Prediction of lymph node metastasis in T1 colorectal cancer based on machine learning

**DOI:** 10.7717/peerj.20500

**Published:** 2026-02-11

**Authors:** Suyujie Shi, Xiongwu Li, Linjun Li, Haowen Zhong, Ruoyan Wang, Zhenyu Zhang, Chuyi Liao, Yun Mao, Meijie Yang, Yaying Yang

**Affiliations:** 1 Department of Clinical Pathology Laboratory of Pathology Diagnostic Center, Chongqing University of Medical Science, Chongqing, China; 2 Department of Pathology, Chongqing University of Medical Science, Chongqing, China; 3 Molecular Medicine Diagnostic and Testing Center, Chongqing University of Medical Science, Chongqing, China; 4 Department of Pathology, The Affiliated Dazu’s Hospital of Chongqing Medical University, Chongqing, China; 5 University of Washington, Seattle, United States of America; 6 School of Basic Medicine, Chongqing University of Medical Science, Chongqing, China; 7 Leicester University Joint College, Chongqing University of Medical Science, Chongqing, China; 8 Department of Radiology, The First Affiliated Hospital of Chongqing University of Medical Science, Chongqing, China; 9 College of Medical Informatics, Chongqing University of Medical Science, Chongqing, China

**Keywords:** Colorectal cancer (CRC), Lymph node metastasis (LNM), Machine learning, Predictive model

## Abstract

**Background:**

Colorectal cancer (CRC) ranks as the third most frequently diagnosed cancer. Early diagnosis and precise risk assessment for lymph node metastasis (LNM) of T1 CRC, characterized by tumor confined to the mucosa and submucosa, essential for enhancing patient outcomes and informing therapeutic strategies. This project aims to use machine learning in refining clinical decision-making processes for T1 CRC patients, thereby laying the groundwork for more personalized and efficacious treatment protocols.

**Methods:**

In this study, we analyzed data from 210 patients with T1 CRC who underwent surgical resection at the First Affiliated Hospital of Chongqing Medical University from 2017 to 2023. The datasets encompassed clinical, endoscopic, and pathological parameters, which were examined to identify potential predictors of LNM. A range of machine learning algorithms, including boosted trees, decision trees, logistic regression, multilayer perceptron (MLP), naïve Bayes, k-nearest neighbors (K-NN), random forest and support vector machine (SVM), were leveraged to construct a predictive model for LNM in T1 CRC.

**Results:**

Our research demonstrated that the random forest algorithm outperformed other models in predictive performance for the risk of LNM. Furthermore, the model identified seven key risk factors associated with LNM. We found four novel LNM predictive indicators for T1 CRC: tumor submucosal invasion area, percentage of tumors with invasive carcinoma, poorly differentiated tumor cell clusters, and serrated lesions.

**Conclusion:**

This study developed a risk predictive model for LNM in T1 CRC patients by utilizing eight machine learning algorithms. Four novel predictive indicators were identified, improving the accuracy of LNM prediction.

## Introduction

Colorectal cancer (CRC) is the third most commonly diagnosed cancer and the second leading cause of cancer-related death globally (Bray et al., 2021; Sung et al., 2021). Early detection, timely diagnosis, and prompt therapeutic intervention are essential for improving patient survival, enhancing quality of life, and prolonging overall survival. The advent of screening programs and advancements in endoscopic diagnostic and resection techniques have significantly increased the detection rate of CRC confined to the mucosa and submucosa (T1). Endoscopic submucosal dissection (ESD) has emerged as an effective treatment option for patients with T1 CRC. Recent studies have shown that ESD can achieve curative outcomes in selected cases of T1 CRC (Libânio et al., 2022; Spaander et al., 2023). In the setting of endoscopic surgery for T1 CRC, if histopathological analysis post-endoscopic resection identifies risk factors associated with lymph node metastasis (LNM), such as submucosal infiltration depth of ≥1,000 µm, lymphatic vascular infiltration, poor differentiation, and high-grade tumor budding, a more extensive surgical procedure may be necessary. This includes a segmental enterectomy, which involves the resection of the affected segment of the intestine, along with a complete lymphadenectomy to remove the regional lymph nodes. However, LNM is present in less than 10% of T1 CRC patients who undergo segmental resection based on the risk stratification criteria outlined in current clinical guidelines, suggesting that additional surgical procedures are often unnecessary (Ouchi et al., 2023). Furthermore, a recent long-term follow-up study on high-risk T1 CRC patients revealed that some individuals, particularly those of advanced age with comorbidities, faced a higher risk of complications and mortality associated with enterectomy and lymph node dissection compared to local tumor resection. It was demonstrated that this more extensive surgical approach did not confer a significant survival benefit (Chen et al., 2023). Therefore, there is an urgent need for more accurate assessment of LNM risk in T1 CRC patients.

Currently, the clinical assessment of LNM risk factors for T1 CRC predominantly relies on histopathological examination. This involves the analysis of hematoxylin and eosin (H&E) stained sections and immunohistochemical analyses of the resected tissue specimens obtained from ESD procedures. However, the assessment of these risk factors by pathologists carries inherent subjectivity, which may lead to inter-individual variability in risk stratification. With the ongoing advancements in artificial intelligence (AI), machine learning (ML) algorithms are becoming increasingly prevalent in the medical field. ML is being integrated into various aspects of healthcare, from diagnostics to treatment planning, with the aim of enhancing the accuracy and efficiency of clinical decision-making (Handelman et al., 2018). It has progressively demonstrated significant utility in the histopathological diagnosis of neoplasms (Wessels et al., 2021). By analyzing digitized whole slide images (WSIs) to identify some objective parameters for LNM in T1 CRC, such as infiltration area, infiltration depth and tumor budding, and constructing machine learning models based on these parameters, the model will become more objective, accurate, and reliable.

In this study, we employed ML algorithms, including boosted trees, decision trees, logistic regression, multilayer perceptron (MLP), naïve Bayes, k-nearest neighbors (K-NN), random forest and support vector machine (SVM) to develop a predictive model for LNM in T1 CRC. This approach aims to improve the accuracy of pathological interpretation, thereby enabling more reliable diagnosis and staging of T1 CRC. By enhancing diagnostic precision, it could reduce unnecessary surgical interventions (particularly those without clear clinical indications) and associated complications. Consequently, this strategy may assist clinicians in developing tailored therapeutic plans for T1 CRC patients, ultimately improving patient treatment outcomes and quality of life, mitigating the overutilization of medical interventions and optimizing healthcare resource allocation.

## Materials and Methods

### Study population

**Figure 1:**
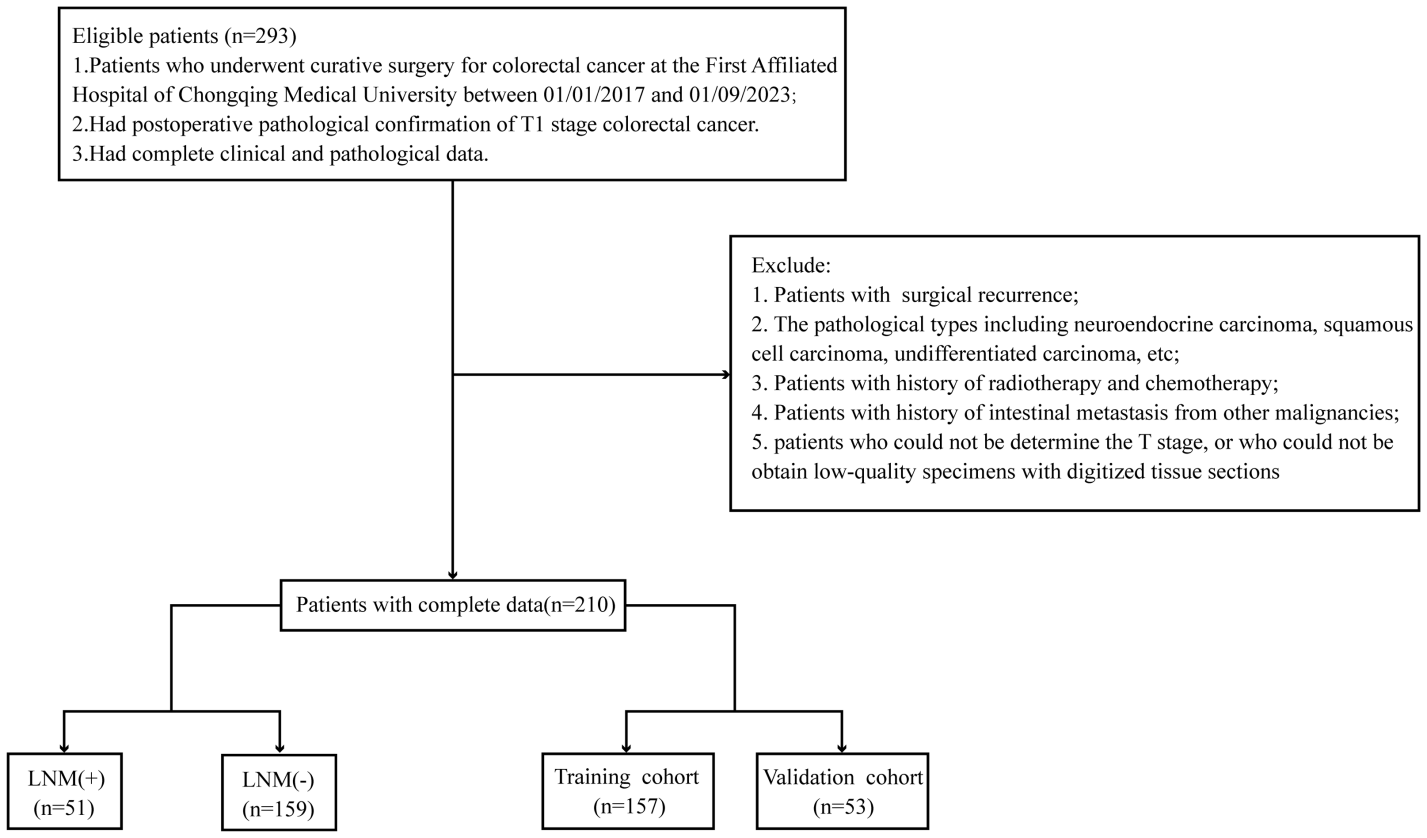
Flow chart showing the selection of study patients.

We collected data from 210 patients diagnosed with T1 CRC who underwent radical resection at the First Affiliated Hospital of Chongqing Medical University between 01/01/2017 and 01/09/2023. We collected the data after obtaining the approval document from the hospital ethics committee. This retrospective study had the following inclusion criteria: (1) Patients who underwent curative surgery (Hohenberger et al., 2009), which is a procedure aimed at complete tumor resection with clear margins and systematic lymphadenectomy to eliminate locoregional metastases for colorectal cancer at the First Affiliated Hospital of Chongqing Medical University between 01/01/2017 and 01/09/2023; (2) had postoperative confirmation of T1 colorectal cancer; (3) had complete clinical and pathological data. Patients with surgical recurrence, the pathological types including neuroendocrine carcinoma, squamous cell carcinoma, undifferentiated carcinoma, *etc*; or with history of radiotherapy and chemotherapy, history of intestinal metastasis of other cancers, patients who could not be determine the T stage, or who could not be obtain low-quality specimens with digitized tissue sections were excluded (Fig. 1).

This study was approved by the Ethics Review Committee of the First Affiliated Hospital of Chongqing Medical University (K2023-345). The requirement for informed consent was waived owing to the retrospective nature of this study.

### Data description

The potential features comprised three categories (Guo et al., 2020; Ichimasa et al., 2021; Xu et al., 2019; Yasue et al., 2019): clinical, endoscopic, and pathological. Clinical features included gender, age, diabetes history, smoking history, blood stool history, body mass index (BMI), carcinoembryonic antigen (CEA) level. Endoscopic features included tumor location, tumor size, and morphology (elevated, flat and depressed). The pathological features included the degree of tumor differentiation, the presence of mucinous carcinoma components, the presence of serrated lesions, the percentage of tumors with invasive carcinoma, the grade of tumor budding, the grade of poorly differentiated tumor cell clusters, the depth of tumor submucosal infiltration, area of tumor submucosal infiltration and lymphatic vascular infiltration.

Tumor budding, defined as clusters of tumor cells, consisting of five or fewer cells, located at the infiltrative front of the tumor.

**Figure 2:**
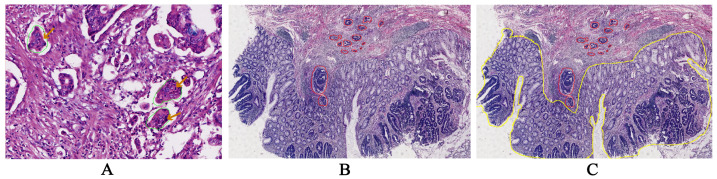
Visual representation of pathological tissue sections. (A) High-grade poorly differentiated tumor cell clusters (marked by the green circle and orange arrow). (B) The area of tumor submucosal infiltration (marked by the red circle, measured by Qupath (0.5.1)). (C) The proportion of infiltration area to the whole tumor area (the whole tumor area marked by the yellow circle, measured by Qupath (0.5.1)).

Poorly differentiated tumor cell clusters, defined as clusters of tumor cells consisting of five or more cells, not forming glandular structures, found at the tumor invasion front and within the tumor stroma (Fig. 2A);

The measurement of submucosal invasion depth is determined as follows (Chinese Society of Oncology, 2023).

• Intact muscularis mucosae: When the muscularis mucosae is histologically identifiable, the depth is measured perpendicularly from its lower border to the deepest invasive tumor front.
• Disrupted muscularis mucosae: In cases where the muscularis mucosae is completely absent, the measurement is taken from the mucosal surface to the deepest submucosal invasive focus, accompanied by stromal desmoplastic reaction.

The submucosal invasive area was quantified as the cumulative sum of all lesional regions between the tumor surface and deepest invasion point (Fig. 2B);

The proportion of infiltration area to the whole tumor area measured by Qupath (v0.5.1), (Fig. 2C). The invasion area ratio was calculated as: (Submucosal invasive area/Total tumor area) ×100%. In cases with complete muscularis mucosae loss, this ratio was defaulted to 100%);

Lymphatic vascular infiltration observed using immunohistochemistry (IHC).

### Machine learning classifiers

The overall dataset was randomly divided into training data set and testing data set in a ratio of 3:1. We utilized eight predictive algorithms, including boosted trees, decision trees, logistic regression, multilayer perceptron (MLP), naïve Bayes, k-nearest neighbors (K-NN), random forest and support vector machine (SVM). To enhance model accuracy and robustness, ten-fold cross-validation was employed. The models’ performance was evaluated using a comprehensive set of metrics: the area under the curve (AUC), specificity, sensitivity, positive predictive value (PPV), negative predictive value (NPV), accuracy, F1 score and receiver operating characteristic curve (ROC). The calibration of the optimal model (random forest) was assessed *via* a calibration curve.Furthermore, decision curve analysis (DCA) was used to evaluate the application value of predictive models in practical clinical decision-making. All analyses were conducted in R language (R 4.2.0; R Core Team, 2022) and Rstudio. The code used in this study is available in a GitHub repository archived on Zenodo (https://doi.org/10.5281/zenodo.17533536).

#### Random forest

Random Forest is an ensemble learning method that operates by combining multiple decision trees to make a collaborative prediction.Briefly, each tree is built using a randomly generated vector Θ_k_, which is independent and identically distributed for all trees. As the number of trees grows, the model’s generalization error stabilizes, influenced by two factors: individual tree accuracy and correlation between trees. During training: Each tree uses a unique Θ_k_ (*e.g.*, bootstrapped data indices in Bagging, random feature subsets in split selection).

The final prediction is determined by majority voting across all the trees in the forest. Throughout this process, the model optimizes splitting criteria at each node to progressively learn data patterns. The core advantage of this approach is that by aggregating a large number of diverse trees, the ensemble significantly reduces the risk of overfitting while maintaining strong predictive performance (generalization ability) on new data, as established in ensemble learning theory (Breiman, 2001; Crimionisi, Shotton & Konukoglu, 2011). Forest training happens by optimizing the parameters of the weak learner at each split node *j via*:

\begin{eqnarray*}{\theta }_{j}^{\ast }=\arg \nolimits {\max \nolimits }_{{\theta }_{j}\in {T}_{j}}{I}_{j}. \end{eqnarray*}

For classification the objective function *I*_*j*_ takes the form of a classical information gain defined for discrete distributions:

\begin{eqnarray*}{I}_{j}=H \left( {S}_{j} \right) -\sum _{i\in \left\{ L,R \right\} } \frac{ \left\vert {S}_{j}^{i} \right\vert }{ \left\vert {S}_{j} \right\vert } H({S}_{j}^{i}) \end{eqnarray*}

with *i* indexing the two child nodes. The entropy for a generic set *S* of training points is defined as:

\begin{eqnarray*}H \left( S \right) =-\sum _{c\in C}p(c)\log \nolimits ~p(c) \end{eqnarray*}

### Statistical analysis

SPSS 25.0 software was used for statistical analysis. Categorical variables are reported as number and percentages, whereas continuous variables are presented as mean ± standard deviation (SD) or median (interquartile range: IQR). Continuous variables conforming to normal distribution were analyzed utilizing Student’s *t*-test Conversely, continuous variables not conforming to normal distribution were analyzed using the Mann–Whitney *U* test. Categorical data were evaluated using the Chi-square test or Fisher’s exact test.

## Results

### Baseline characteristics of the study population

A total of 293 patients with T1 CRC were eligible for this study. After excluding 83 patients based on our exclusion criteria, 210 patients were finally analyzed. Table 1 demonstrates the clinical and pathological characteristics of the lymphatic node metastasis group (*n* = 51) and the lymph node non-metastasis group (*n* = 159). Statistically significant (*P* < 0.05) differences were observed with tumor submucosal infiltration area, percentage of tumors with invasive carcinoma, degree of differentiation, tumor budding, lymphatic and vascular invasion, mucinous carcinoma component, submucosal infiltration depth, high-grade poorly differentiated tumor cell clusters and serrated lesions between the two groups.

**Table 1:** Baseline characteristics of the study population.

	All patients (*n* = 210)	Lymphatic node metastasis (*n* = 51)	Lymph node non-metastasis (*n* = 159)	*P* value
Gender				0.743
Male	128(61.0%)	30(58.8%)	98(61.6%)	
Female	82(39.0%)	21(41.2%)	61(38.4%)	
Age	210	61.3 ± 11.3	64.0 ± 11.2	0.150
BMI^a^	210	22.6 ± 2.8	23.1 ± 3.0	0.291
Tumor submucosal infiltration area	210	32.9(18.5,54.8)	20.1(10.4,30.2)	0.001^*^
Percentage of tumors with invasive carcinoma	210	68.6(43.8,84.7)	38.8(16.8,64.8)	0.001^*^
Tumor size	210	2.0(1.5,3.0)	2.0(1.5,3.0)	1.701
CEA^b^	210	2.3(1.7,3.5)	2.1(1.4,3.2)	0.105
Degree of differentiation				0.001^*^
Low	7(3.3%)	6(11.7%)	1(0.6%)	
Medium	165(78.6%)	42(82.4%)	123(77.4%)	
High	38(18.1%)	3(5.9%)	35(22%)	
Lymphatic and vascular invasion				0.001^*^
Yes	31(14.7%)	26(50.9%)	6(3.8%)	
No	179(85.3%)	25(49.1%)	153(96.2%)	
Tumor budding				0.001^*^
Grade 2 or 3	25(11.9%)	21(41.2%)	4(2.5%)	
Grade 1	185(88.1%)	30(58.8%)	155(97.5%)	
Submucosal infiltration depth				0.023
≥ 1,000 μm	171(81.4%)	47(92.1)	124(77.9%)	
<1,000 μm	39(18.6%)	4(7.9%)	35(22.1%)	
Location				0.403
Ascending colon	25(11.9%)	4(7.8%)	21(13.2%)	
Transverse colon	18(8.6%)	2(3.9%)	16(10.1%)	
Descending colon	7(3.3%)	2(3.9%)	5(3.1%)	
Sigmoid colon	29(13.8%)	6(11.8%)	23(14.4%)	
Rectum	131(62.4%)	37(72.6%)	94(59.2%)	
Mucinous carcinoma component				0.001^*^
Yes	16(7.6%)	11(21.6%)	5(3.1%)	
No	194(92.4%)	40(78.4%)	154(96.9%)	
High-grade poorly differentiated tumor cell clusters				0.001^*^
Yes	30(14.3%)	26(50.9%)	4(2.5%)	
No	180(85.7%)	25(49.1%)	155(97.5%)	
Serrated lesions				0.001^*^
Yes	15(7.1%)	11(21.6%)	4(2.5%)	
No	195(92.9%)	40(78.4%)	155(97.5%)	
Blood stool history				0.059
Yes	159(75.7%)	44(86.3%)	115(72.3%)	
No	51(24.3%)	7(13.7%)	44(27.7%)	
Morphology				0.11
Elevated	103(49.1%)	33(64.8%)	98(61.2%)	
Flat	93(44.3%)	12(23.5%)	53(33.7%)	
Depressed	14(6.6%)	6(11.7%)	8(5.1%)	
Diabetes history				0.575
Yes	19(8.9%)	3(5.9%)	16(11.2%)	
No	191(91.1%)	48(94.1%)	143(98.8%)	
Smoking history				0.827
Yes	56(26.7%)	13(25.5%)	43(26.9%)	
No	154(73.3%)	38(75.5%)	116(73.1%)	

**Notes.**

a BMI, Body Mass Index.

b CEA, Carcinoembryonic Antigen.

* *P* < 0.05.

### Characteristics of training and testing cohorts

A total of 210 patients were randomly split into the training cohorts and the testing cohorts at a ratio of 3:1. LNM was found in 38 (24.2%) and 13 (24.5%) patients in the training and testing cohorts, respectively. Characteristics of training and testing cohorts are shown in Table 2. There was no significant difference in clinical data between the two groups (*P* > 0.05).

**Table 2:** Characteristics of training and testing cohorts.

	Validation cohorts (*n* = 53)	Training cohorts (*n* = 157)	*P*
Gender			0.921
Male	21 (39.6%)	61 (38.9%)	
Female	32 (60.4%)	96 (61.1%)	
Age	63.2 (11%)	63.4 (11.5%)	0.906
Degree of differentiation			0.759
Low	1 (1.9%)	6 (3.8%)	
Medium	43 (81.1%)	122 (77.7%)	
High	9 (17%)	29 (18.5%)	
Lymphatic and vascular invasion			0.414
No	47 (88.7%)	132 (84.1%)	
Yes	6 (11.3%)	25 (15.9%)	
Tumor budding			0.257
Grade 1	49 (92.5%)	136 (86.6%)	
Grade 2 or 3	4 (7.5%)	21 (13.4%)	
Submucosal infiltration depth			0.636
<1,000 μm	11 (20.8%)	28 (17.8%)	
≥ 1,000 μm	42 (79.2%)	129 (82.2%)	
Location			0.879
Ascending colon	7 (13.2%)	18 (11.5%)	
Transverse colon	4 (7.5%)	14 (8.9%)	
Descending colon	1 (1.9%)	6 (3.8%)	
Sigmoid colon	9 (17%)	20 (12.7%)	
Rectum	32 (60.4%)	99 (63.1%)	
Mucinous carcinoma component			0.557
No	48 (90.6%)	146 (93%)	
Yes	5 (9.4%)	11 (7%)	
High-grade poorly differentiated tumor cell clusters			0.476
No	47 (88.7%)	133 (84.7%)	
Yes	6 (11.3%)	24 (15.3%)	
Tumor size	2 (1.5,3)	2 (1.5,2.5)	0.347
Percentage of tumors with invasive carcinoma	43.8 (20.8,68.9)	45.3 (20.6,72.6)	0.815
Serrated lesions			0.765
No	50 (94.3%)	145 (92.4%)	
Yes	3 (5.7%)	12 (7.6%)	
Blood stool history			0.747
No	12 (22.6%)	39 (24.8%)	
Yes	41 (77.4%)	118 (75.2%)	
BMI^a^	22.9 (2.9%)	23 (3%)	0.808
Tumor submucosal infiltration area	23.5 (13.7,35.6)	21.8 (12.3,33.6)	0.828
CEA^b^	2.1 (1.5,3)	2.2 (1.4,3.3)	0.847
Morphology			0.198
Elevated	34(64.1%)	85 (54.1%)	
Flat	18(34%)	59(37.6%)	
Depressed	1 (1.9%)	13 (8.3%)	
Diabetes history			0.267
No	46 (86.8%)	145 (92.4%)	
Yes	7 (13.2%)	12 (7.6%)	
Smoking history			0.756
No	38 (71.7%)	116 (73.9%)	
Yes	15 (28.3%)	41 (26.1%)	
Lymph node metastasis			0.962
Negative	40 (75.5%)	119 (75.8%)	
Positive	13 (24.5%)	38 (24.2%)	

**Notes.**

a BMI, Body Mass Index.

b CEA, Carcinoembryonic Antigen.

### Prediction performances of eight different ML algorithms

**Figure 3:**
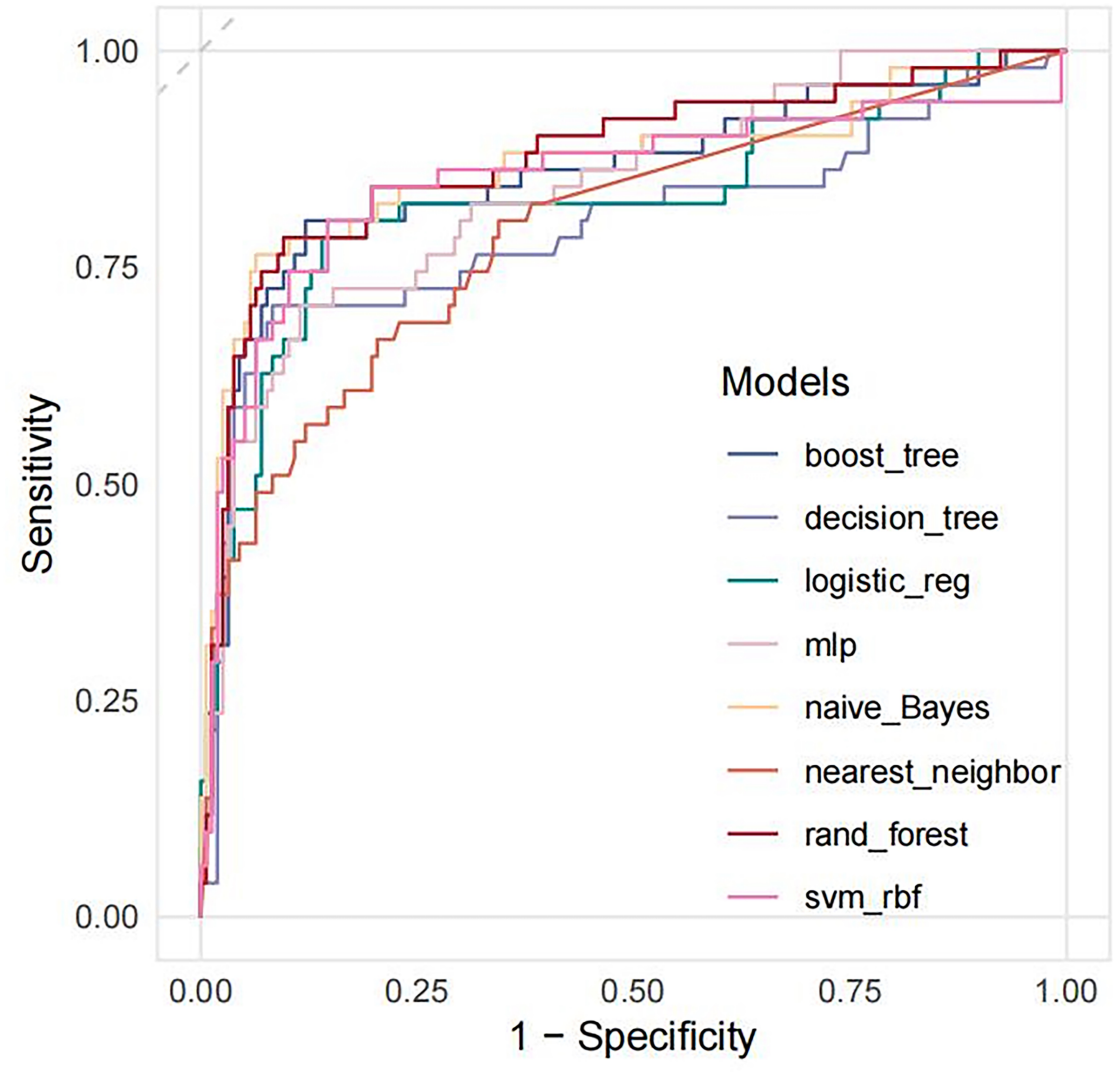
The comparison of ROC curves of eight different ML.

**Figure 4:**
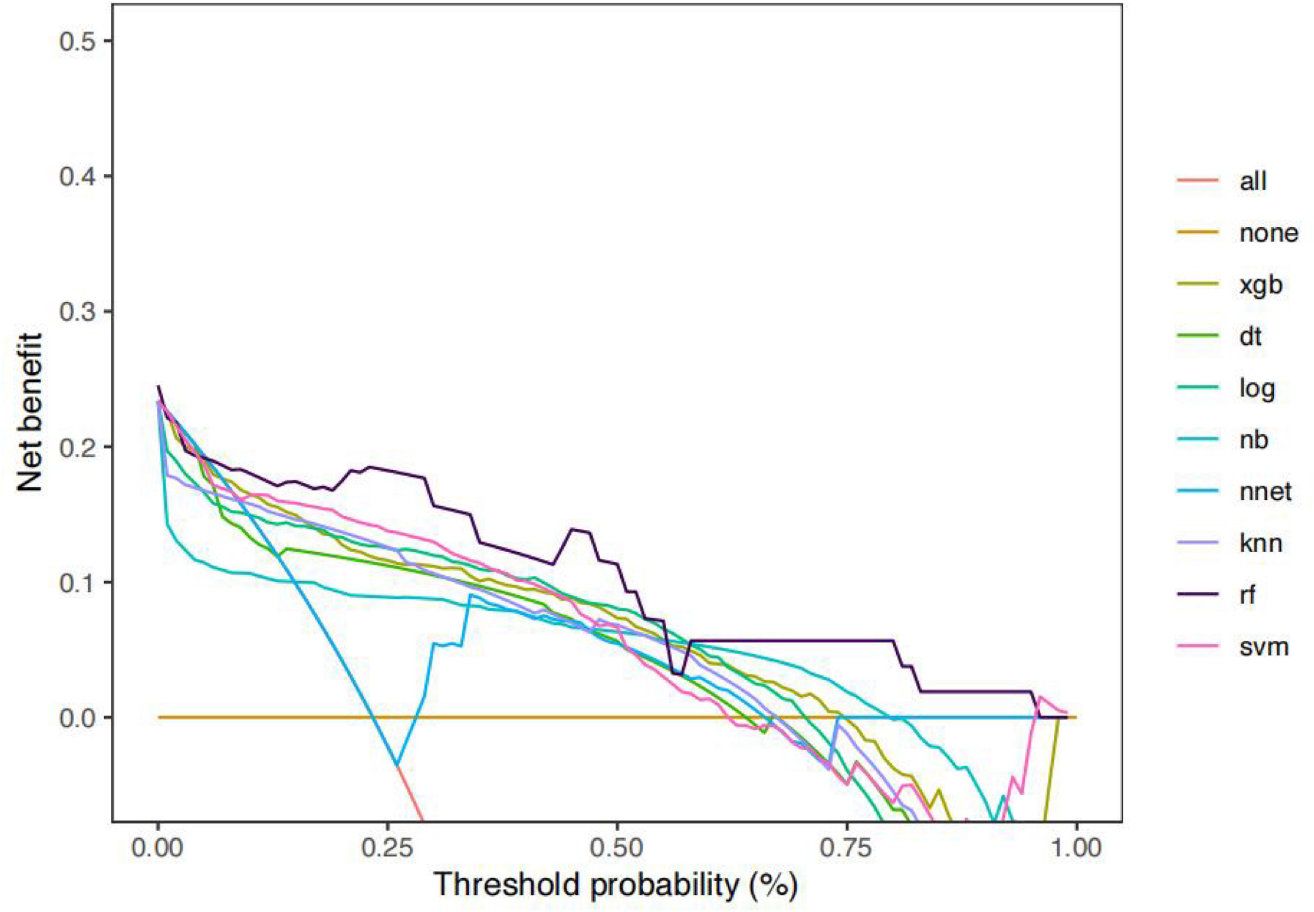
The comparison of DCA curves of eight different ML.

**Figure 5:**
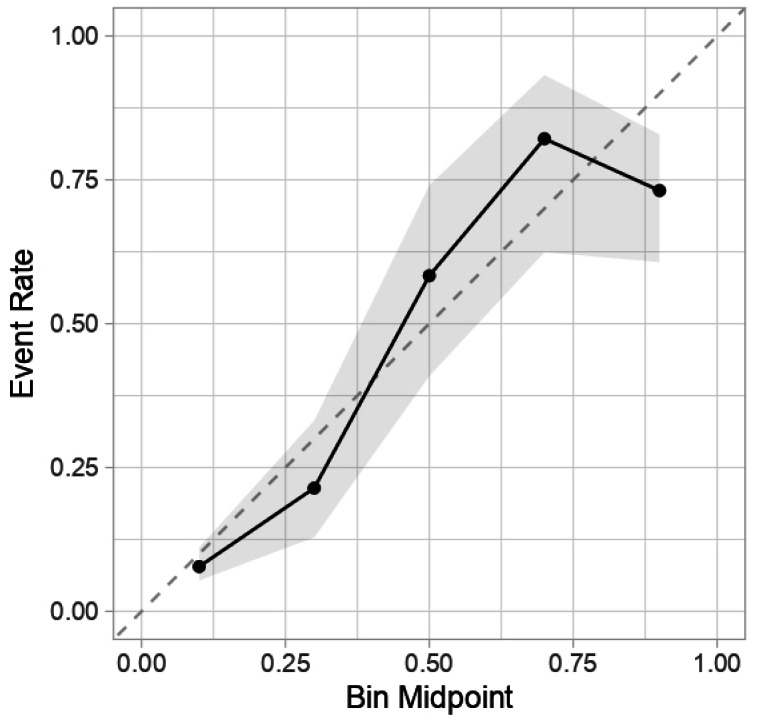
Random forest model calibration curve chart.

The accuracy, sensitivity, specificity, AUC, PPV, NPV and F1 scores of LNM prediction for T1 CRC using eight machine learning methods are shown in Table 3. Based on the evaluation metrics, our results indicate that the random forest model demonstrates the best predictive performance among the models assessed. ROC curves and DCA curves of the models are shown in Figs. 3 and 4. The calibration curve for the optimal random forest model is presented in Fig. 5.

**Table 3:** Prediction performances of eight different ML algorithms.

Algorithms	Accuracy	Sensitivity	Specificity	AUC (95%CI)	PPV^a^	NPV^b^	F1
Boosted trees	0.837	0.774	0.780	0.837(0.7875–0.8747)	0.517	0.919	0.620
Decision trees	0.753	0.584	0.904	0.735(0.6896–0.7958)	0.650	0.877	0.615
Logistic regression	0.791	0.672	0.889	0.791(0.7145–0.8229)	0.648	0.899	0.659
MLP^c^	0.847	0.730	0.904	0.847(0.7726–0.8697)	0.699	0.916	0.714
Naïve Bayes	0.723	0.591	0.895	0.731(0.671–0.7832)	0.633	0.878	0.611
KNN^d^	0.807	0.730	0.824	0.807(0.7654–0.8532)	0.559	0.909	0.633
Random forest	0.872	0.737	0.900	0.872(0.8158–0.8953)	0.692	0.918	0.714
SVM^e^	0.819	0.774	0.844	0.825(0.7561–0.8532)	0.602	0.924	0.677

**Notes.**

a PPV, Positive Predictive Value.

b NPV, Negative Predictive Value.

c MLP, Multilayer Perceptron.

d KNN, K-Nearest Neighbors.

e SVM, Support Vector Machine.

### Analyze the influencing factors

**Figure 6:**
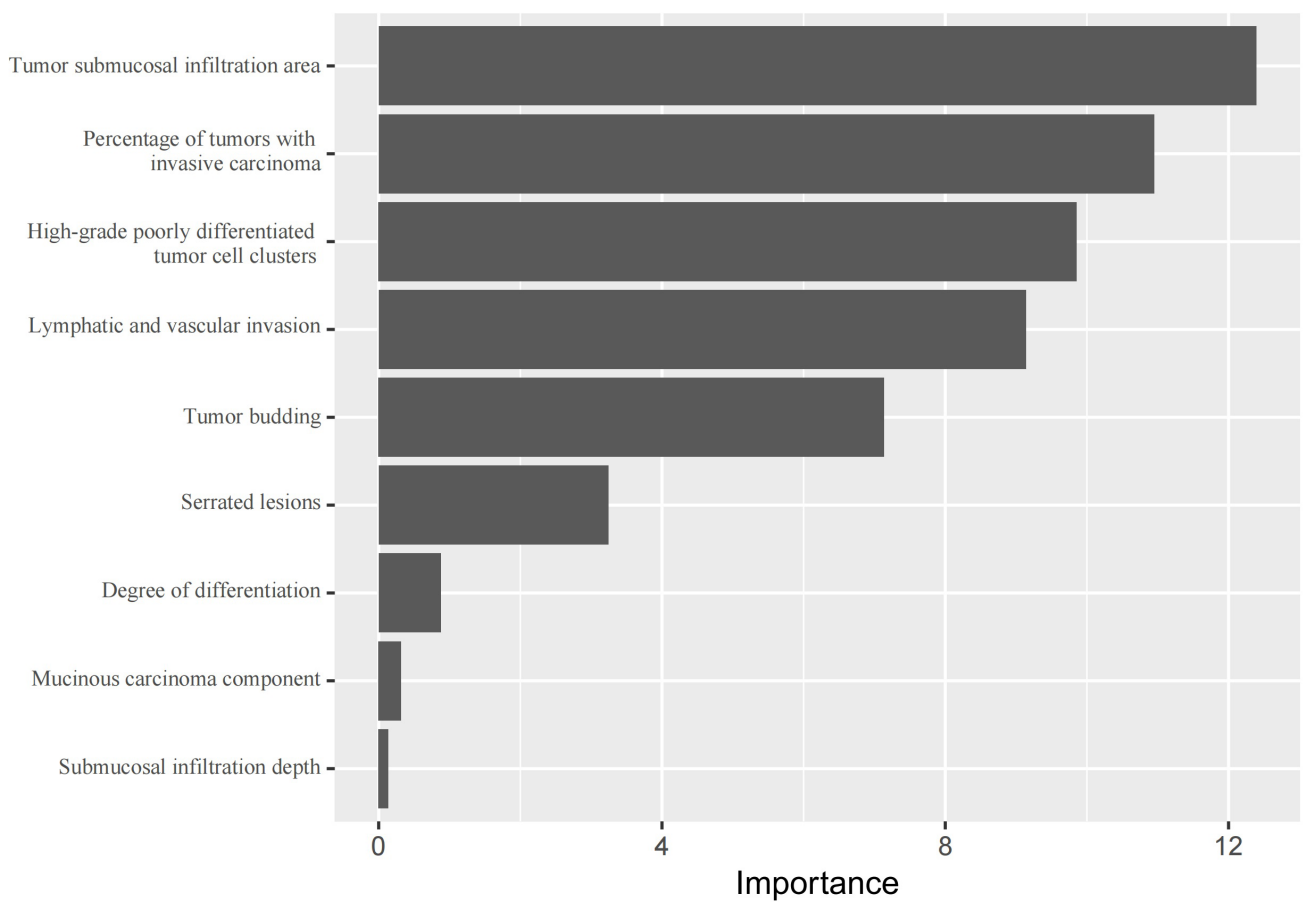
Variable importance ranking of the random forest model. Features are ranked from top to bottom by their importance score. Higher value indicates a greater contribution to the model’s predictive performance. ‘Tumor submucosal infiltration area’ was identified as the most influential predictor, followed by ‘Percentage of tumors with invasive carcinoma’ and ‘High-grade poorly differentiated clusters’.

The top five important variables using random forest were as follows: tumor submucosal infiltration area, percentage of tumors with invasive carcinoma, high-grade poorly differentiated tumor cell clusters, lymphatic and vascular invasion and tumor budding (as shown in Fig. 6).

## Discussion

ESD is the primary treatment for T1 CRC. However, whether patients who undergo endoscopic resection require further bowel resection and lymph node dissection depends on the pathological evaluation of the resected specimen for the risk of LNM. In clinical practice in China, Japan, and Western countries, similar criteria are used to assess the risk of LNM (Benson et al., 2021; Hashiguchi & Saito, 2020; Libânio et al., 2022). High-risk factors for LNM are identified in cases where the endoscopic resection specimen demonstrates the following characteristics: (1) Poor prognostic histological features, such as poorly differentiated adenocarcinoma, signet-ring cell carcinoma, or mucinous adenocarcinoma; (2) vascular or lymphatic invasion; (3) incomplete resection, fragmented specimen, or inability to assess the resection margins; (4) submucosal invasion depth ≥ 1,000 µm; (5) positive resection margins; (6) high-grade tumor budding (grade 2/3) (Chinese Society of Oncology, 2023). According to current guidelines, over 90% of patients with T1 CRC do not exhibit LNM after undergoing bowel resection (Tanaka et al., 2024), suggesting that the existing LNM risk assessment methods may not be sufficiently accurate. The predictive model developed in this study innovatively employs single-factor risk indicators for construction, compared to traditional multifactorial assessment approaches. It demonstrates enhanced predictive accuracy performance in evaluating LNM risk.

Current LNM risk assessment methods for patients with T1 CRC include relatively few predictive parameters for high-risk LNM. Additionally, some of these parameters, such as lymphovascular invasion and degree of differentiation, rely on subjective interpretation by pathologists, leading to relatively low inter-observer agreement (Ichimasa et al., 2022). Therefore, the accuracy of current guidelines in predicting LNM in T1 CRC is limited. To reduce unnecessary surgical interventions, alleviate the treatment burden on patients, and minimize the waste of medical resources, it is essential to identify more accurate and objective risk factors and establish more effective predictive models to guide clinical treatment. Predictive models, which integrate multiple variables such as clinical indicators, biochemical markers, and imaging data, aim to predict the probability of a specific outcome, helping clinicians to more accurately assess the risk and prognosis of patients, thus enhancing clinical decision-making and providing personalized medical care (Moons et al., 2009; Steyerberg et al., 2013). With the continuous advancement of artificial intelligence (AI) technology, machine learning (ML) algorithms are playing an increasingly important role in cancer diagnosis and prognosis evaluation (Handelman et al., 2018). Several studies have already developed predictive models for LNM in T1 CRC (Fujino et al., 2023; Kajiwara et al., 2023; Kang et al., 2021; Oh et al., 2019). For example, a predictive model based on clinical-pathological parameters established using Least Absolute Shrinkage and Selection Operator (LASSO) regression has demonstrated good performance (Kang et al., 2021); similarly, a model using the random forest algorithm to predict LNM based on histological analysis of pathological slides has been evaluated using AUC as a measure of model performance (Takamatsu et al., 2022). However, for AI-driven predictive models to be truly useful in clinical practice, high discriminative performance alone is insufficient (Vasey et al., 2022). The calibration of a model—the agreement between predicted probabilities and observed outcomes—is equally critical for clinicians to trust and act upon probabilistic predictions (Vasey et al., 2022). Furthermore, existing models predominantly rely on restricted feature sets or single-algorithm frameworks, limiting their accuracy and generalizability.

In this study, we collected clinical, endoscopic, and pathological data of 20 parameters from patients with T1 CRC. To identify the model with the optimal performance, we applied and compared eight different machine learning algorithms (including boosted trees, decision tree, logistic regression, MLP, naïve Bayes, K-NN, random forest and SVM) to construct and benchmark predictive models. In this study, 10-fold cross-validation was performed during the training process for each of the eight machine learning algorithms. The performance of each model was evaluated based on accuracy, sensitivity, specificity, AUC, PPV, NPV, and F1 score. The random forest algorithm was ultimately identified as the optimal predictive model for LNM in T1 CRC. Notwithstanding its performance, the clinical translation of machine learning models such as this necessitates careful consideration of interpretability and integration challenges. Unlike conventional histopathological examination, which provides direct morphological evidence, ML models generate probabilistic predictions without inherent explainability. This “black-box” nature may impede clinical trust and adoption, as pathologists and surgeons require understandable rationale for decision-making (King et al., 2023; Linardatos, Papastefanopoulos & Kotsiantis, 2020). Therefore, the proposed model should be positioned as a decision-support tool that complements, rather than replaces, standard histopathological assessment. For example, we envision this model being integrated into Electronic Medical Record (EMR) systems to provide immediate, data-driven risk assessments for multidisciplinary team discussions and shared decision-making. Future work should focus on implementing explainability techniques and optimizing this kind of workflow integration to facilitate clinical adoption.

Relevant studies have shown that the risk factors for metastasis and recurrence in T1 CRC, as well as the associated risk Predictive models, identify factors such as lymphovascular invasion, high-grade tumor budding, and tumor size >1 cm, *etc.*, which are highly correlated with LNM (Xu et al., 2020). In this study, by analyzing the variable importance in random forest model, we identified four additional predictive factors beyond the conventional LNM indicators: tumor submucosal invasion area, percentage of tumors with invasive carcinoma, high-grade poorly differentiated tumor cell clusters, and serrated lesions. The four novel indicators proposed in this study may provide more comprehensive predictive factors for clinicians and pathologists, thereby improving the accuracy of predictive model for LNM risk in T1 CRC patients.

In previous studies, a submucosal invasion depth of ≥1,000 µm, rather than the area of submucosal invasion, has been considered a high-risk factor for LNM in T1 CRC and a strong indication for surgical resection. However, a meta-analysis has shown that when submucosal invasion depth ≥1,000 µm is the sole factor, the absolute risk of LNM in T1 CRC is only 2.6% (Zwager et al., 2022). Other researchers, through three-dimensional studies of colorectal cancer mucosa, suggested that the area of submucosal invasion could replace the depth of invasion in assessing the risk of LNM (Toh et al., 2015), and proposed that an invasion area >35 mm^2^ indicates a significantly higher risk of LNM. In our study, we first identified, through univariate regression analysis, that the tumor’s submucosal invasion area is significantly positively correlated with the LNM risk in T1 CRC. Furthermore, we also confirmed this finding in our predictive model. Additionally, tumor invasiveness is closely associated with LNM. T1 CRC often develops from adenomas or serrated lesions, and therefore, invasive carcinoma may coexist with adenomatous or serrated components within the tumor. In both univariate regression and our predictive model, this study confirmed that a higher percentage of invasive carcinoma within the entire tumor is associated with an increased risk of LNM. Tumor budding refers to isolated, undifferentiated tumor cells or small clusters (up to four cells) scattered at the invasive front of the tumor. High-grade tumor budding is currently considered a high-risk factor for LNM according to guidelines. In our study, this conclusion was also confirmed through univariate regression analysis and the development of a machine learning algorithm model. Recently, it has been found that poorly differentiated clusters, consisting of five or more tumor cells forming small aggregates, are closely related to the malignant biology of CRC. These clusters have been considered to represent a manifestation of epithelial-mesenchymal transition (EMT), similar to tumor budding. As a result, poorly differentiated clusters have been proposed as an independent prognostic factor for CRC (Kim, Shin & Kim, 2015). Additionally, studies have shown that high-grade poorly differentiated clusters are significantly associated with the KRAS mutation status in CRC, which correlates with deeper wall invasion and LNM. This may help explain the poorer prognosis of *KRAS*-mutated CRC (Barresi, Reggiani Bonetti & Bettelli, 2015; Makrodouli et al., 2011). In our study, we incorporated poorly differentiated clusters into the analysis, and both univariate regression and predictive models demonstrated that poorly differentiated clusters are indeed associated with the risk of LNM in T1 CRC, serving as an important predictive indicator of LNM. Serrated lesions of the colon and rectum are considered precursor lesions of CRC and are closely linked to microsatellite instability (MSI) and the CpG island methylator phenotype (CIMP) (Aiderus, Barker & Tergaonkar, 2024; Nagtegaal et al., 2020). It is currently believed that, compared to the classic adenoma-carcinoma pathway, the BRAF^V600E^ mutation is a key initiating factor driving serrated lesions (Jones et al., 2017; Tang et al., 2024). More than 70% of patients with the BRAF^V600E^ mutation exhibit microsatellite instability (MSI-H), with tumors predominantly presenting as mucinous carcinoma, poor differentiation, and poor prognosis. Therefore, we speculate that the presence of serrated lesions often suggests that the pathogenesis of CRC in these patients is associated with the serrated pathway, potentially leading to a worse clinical prognosis. In our observation of case slides, we found that approximately 7% of the cancer tissues contained serrated lesions, and these serrated lesions were shown to be a high-risk factor for LNM in patients with T1 CRC.

## Conclusion

In summary, this study developed a risk predictive model for LNM in patients with T1 CRC using eight machine learning algorithms, which enhanced the accuracy of LNM prediction. However, there are certain limitations in this study, including the relatively homogeneous data sources and the limited sample size. In future research, we plan to validate and refine the model using data from multiple centers and larger cohorts, with the aim of further improving its generalizability and clinical applicability. Ultimately, this would provide a more precise risk assessment tool for individualized treatment of CRC patients.

##  Supplemental Information

10.7717/peerj.20500/supp-1 Supplemental Information 1 Raw data

10.7717/peerj.20500/supp-2 Supplemental Information 2 Code

10.7717/peerj.20500/supp-3 Supplemental Information 3 STROBE checklist

## Additional Information and Declarations

### Data Availability

The following information was supplied regarding data availability: The raw data and code are available in the Files S1 and and S2. In addition, we have shared the code on GitHub and have deposited the GitHub repository in Zenodo, where it has been assigned a permanent DOI: https://doi.org/10.5281/zenodo.17533536.
